# A topology-based evaluation of resilience on urban road networks against epidemic spread: Implications for COVID-19 responses

**DOI:** 10.3389/fpubh.2022.1023176

**Published:** 2022-10-18

**Authors:** Junqing Tang, Huali Lin, Xudong Fan, Xiong Yu, Qiuchen Lu

**Affiliations:** ^1^School of Urban Planning and Design, Peking University, Shenzhen Graduate School, Shenzhen, China; ^2^Key Laboratory of Earth Surface System and Human-Earth Relations of Ministry of Natural Resources of China, Shenzhen Graduate School, Peking University, Shenzhen, China; ^3^Department of Civil and Environmental Engineering, Case Western Reserve University, Cleveland, OH, United States; ^4^The Bartlett School of Sustainable Construction, University College London, London, United Kingdom

**Keywords:** transport resilience, road networks, epidemic spreading, COVID-19, emergency management

## Abstract

Road closure is an effective measure to reduce mobility and prevent the spread of an epidemic in severe public health crises. For instance, during the peak waves of the global COVID-19 pandemic, many countries implemented road closure policies, such as the traffic-calming strategy in the UK. However, it is still not clear how such road closures, if used as a response to different modes of epidemic spreading, affect the resilient performance of large-scale road networks in terms of their efficiency and overall accessibility. In this paper, we propose a simulation-based approach to theoretically investigate two types of spreading mechanisms and evaluate the effectiveness of both static and dynamic response scenarios, including the sporadic epidemic spreading based on network topologies and trajectory-based spreading caused by superspreaders in megacities. The results showed that (1) the road network demonstrates comparatively worse resilient behavior under the trajectory-based spreading mode; (2) the road density and centrality order, as well as the network's regional geographical characteristics, can substantially alter the level of impacts and introduce heterogeneity into the recovery processes; and (3) the resilience lost under static recovery and dynamic recovery scenarios is 8.6 and 6.9%, respectively, which demonstrates the necessity of a dynamic response and the importance of making a systematic and strategic recovery plan. Policy and managerial implications are also discussed. This paper provides new insights for better managing the resilience of urban road networks against public health crises in the post-COVID era.

## 1. Introduction

Since 2020, the global COVID-19 pandemic has not only triggered major shifts in city operations, but has also had a considerable impact on people's travel behaviors, as well as their abilities to work and their mental health (1–4). Many countries have adopted strict public health control measures to contain the spread of this unprecedented and highly infectious virus, such as travel restrictions, road closures, and social distancing. While road closures, for instance, are considered effective in isolating certain high-risk areas and communities so that the infectious spread may be quickly controlled, such “no access” restriction measures could substantially harm not only the local accessibility and road network connectivity but also many other unexpected aspects of our daily life. For example, a recent study on the impact of COVID-19 related “stay-at-home” restrictions on food prices in Europe showed that vegetable prices increased by 3.36% in the high restriction group compared to the low-restriction group (5). Despite the knowledge of the general impacts of the access control measures, there is still very little known about how those strict control measures, as responses to the various types of virus spreading, could affect the resilience performance of the road networks in megacities. More importantly, investigation on this topic might reveal useful implications for better preparing for the challenges in the post-COVID time.

The key theme this paper is focusing here is about the resilience of the urban road networks, and this topic has been extensively studied by many scholars, under the context of various types of natural and man-made disasters. However, studies on the resilience of urban road networks against infectious diseases have been relatively limited, which leads to a lack of knowledge on building resilient urban transportation systems in the context of emergency response management for public health crises. Such scarcity is even more prominent when considering various types of contagious spreading mechanisms including the sporadic and trajectory-based occurrence of the disease. The former refers to the cases that are scattered or are in a small cluster and separated in place so that little or no connection exists among them, and more importantly, those cases also do not show a recognizable common source. The latter is also known as the superspreader spreading mode. A superspreader is often considered an infected individual who has infected others disproportionately (6, 7). In this paper, we adopt the definition as those who infect many others along their trajectories of movement. Furthermore, from the perspective of quickly recovering the systemic serviceability in emergency responses, it is also not very clear how much dynamic recovery strategies outperform static recovery strategies under the two abovementioned spreading mechanisms. Addressing these unclear questions has both practical and managerial implications for a better urban management. As a result, the objectives of this paper can be summarized as follows.

1. To conduct an in-depth assessment of resilience on urban road networks against the two aforementioned epidemic spreading mechanisms and reveal their dissimilar negative effects in terms of the performance loss;
2. To perform comparative studies between the static and dynamic recovery strategies and quantitatively reveal the effectiveness and shortcomings in terms of the performance restoration.

To achieve the objectives, this paper conducts a simulation-based comparative investigation to understand the epidemic spreading and the recovery processes on urban road networks. Taking Beijing, China, as an illustrative case study, we used the classic susceptible-infectious-recovery (SIR) model and network analytics to simulate the *sporadic occurrence* and *trajectory-based occurrence*. Additionally, we comparatively analyze the performance of urban road networks under two recovery strategies, namely the “*First-Close-First-Reopen” (FCFR)* recovery strategy and the *dynamic* recovery strategy. The resilience, in terms of performance loss, from all cases is evaluated to quantitatively benchmark their heterogeneous effectiveness, which yields several critical implications for practical COVID-19 emergency responses in megacities. The main contributions of this paper can be summarized as following:

- This paper quantitatively measures the advantages of the dynamic road recovery strategy over the widely applied static one under the context of various epidemic spreading mechanisms, providing additional new evidence and insights for relevant decision makers and stakeholders.
- This study lays a foundation for better understanding the impacts of the emergency road closures (either temporary or permanent) on the efficiency of road networks, which is useful for local authorities to manage more efficient responses to future public health crises in urban emergency management.
- To the best of our knowledge, this paper is one of the earliest studies that discuss the superspreader spreading mechanism in the field of transportation resilience, which fills a clear gap for the development of the frontier.

The remainder of this paper is organized as follows: Section 2 reviews the state-of-the-art knowledge on two resilience-related topics, namely resilience assessment and infectious spreading on urban roads, and identifies and further clarifies the knowledge gaps and missing links. Section 3 describes the proposed simulation-based analytical approach and the applied methods, including details on two spreading mechanisms and two recovery strategies. Section 4 outlines the essential background information about the case study and data. Section 5 presents the main findings of this investigation. Then, Section 6 discusses and summarizes several managerial implications, followed by the conclusion and future research directions of the paper in Section 7.

## 2. Literature review

### 2.1. Assessment of resilience on urban road networks

The resilience of urban road networks is crucial to the operation of modern cities, especially when disruptions occur and prompt responses are needed. This particular stream of research occupies a large share of the resilience-related fields in infrastructure studies. Many studies discussed the assessment of road network resilience against natural disasters such as earthquakes, floods, landslides, heavy snow, etc. For example, Aydin et al. (8) developed a method to assess the resilience of urban road networks under seismic hazards using graph theory and stress testing methods, and Zhou et al. (9) studied the connectivity of post-earthquake road networks by using percolation based method. Gao et al. (10) used the Bayesian network (BN) as a modeling tool to assess road network resilience and component importance under different earthquake magnitudes, which showed that the higher the earthquake level is, the lower the system resilience. Morelli and Cunha (11) proposed a novel method for measuring urban road resilience against floods based on travel distribution, and Zhang et al. (12) quantitatively assessed the vulnerability and resilience of urban traffic under different rainfall intensities. Aside from those previous examples focusing on single events, some scholars approached the issue from the perspective of multiple hazards. Der Sarkissian et al. (13) assessed road resilience to different natural hazards with a developed network analytic method and Zhou et al. (14) developed a novel two-layer framework to assess the robustness of transportation networks considering multiple hazard events. Additionally, there are many studies on the resilience of road networks, readers can refer to some recent studies in this field such as Zhou et al. (15) and Serdar et al. (16).

From the above, resilience assessment highly hinges on different scenarios, causing difficulties in simulation-oriented studies. Recovery modeling and simulation on different infrastructure networks, such as interdependent utility networks and electric power networks, have been not uncommon in the relevant fields (17–23). For example, Li et al. (24) simulated the effect of road closures caused by pluvial flash floods in multiple scenarios with a GIS-based model. Wang and Liu (25) proposed a mathematical model for measuring the recovery of urban road networks in snow events and established snow removal resource location and allocation optimization models to resolve the issue. In addition, some studies also introduced other methods to discuss the simulation recovery on road networks. For instance, Vodák et al. (26) introduced a modified ant colony optimization algorithm to study the recovery process of road networks after disasters, which can be used for planning construction work when damage occurs. Zhan et al. (27) employed traffic congestion on road networks as a case study and proposed a new framework for modeling the evolution of functional failures and recoveries in complex road networks. Sohouenou and Neves (28) compared the effects of several link-repair strategies on road network resilience across a multitude of perturbation scenarios and analyzed the characteristics of the optimal recovery strategy. The findings showed that it was important to consider and model the recovery processes for critical disruption scenarios that affect a large number of links. In addition, Tang et al. (29) tested the effect of different sensor recovery schedules on the resilience of traffic-sensor networks through the analysis of the spatial-temporal vehicle patterns in Cambridge, UK. The simulation results suggested that a prioritized sensor maintenance recovery plan would enable more efficient use of public resources.

### 2.2. Infectious spreading on urban roads

The spread of infectious diseases has had great impacts on the public health and transportation sectors and thus, has been well-documented in many previous studies (30–33). The rapid development of the urban built environment and transportation infrastructure can facilitate the spread of infectious diseases and amplify their scale (34). For years, numerous efforts have been dedicated to building models for studying infectious diseases in public transportation systems. For example, Qian and Ukkusuri (21) developed a novel Trans-SEIR modeling approach to connect urban transportation systems with the spread of infectious diseases. The results can guide the optimal placement of entrance control over the public transportation system, such as buses and metros, and thus, help to mitigate the risks of infectious diseases. Mo et al. (35) proposed a time-varying weighted public transit (TWPT) encounter network to model disease spreading through transit systems, considering social activity contacts at both local and global levels. The results showed that early identification and isolation of infected passengers can effectively reduce the spread. Using the susceptible-or-infected (SI) and susceptible-infected-recovery (SIR) models, Chatterjee et al. (36) studied the dynamic process of epidemic outbreaks and information diffusion on urban bus networks in six Indian cities. They discovered that the characteristic path length is vital for information diffusion and epidemic spreading. In addition, Zhang et al. (37) analyzed the transmission mechanism of the COVID-19 epidemic along traffic routes based on population migration, using an improved SEIR model.

Moreover, a few studies have also addressed the relationship between transportation systems and several specific diseases such as dengue and tuberculosis. Li et al. (38) studied the spatial and temporal changes in dengue spread and its spatial relationship with road networks in southern Chinese cities. Their results indicated that cases were concentrated near narrow roads and that the epidemic spread mainly along high-density road network areas, which partially explains the underlying mechanism of the occurrence of sporadic epidemic hotspots during the early spreading stage. Ge et al. (39) examined the association between tuberculosis (TB) incidence and four types of transportation networks at the provincial level, identifying spatial clusters of TB incidence linked with transportation networks in different regions. COVID-19 has been a popular research focus in transportation studies in recent years (40, 41). Among them, analyzing the control measures and policy implications is of great value for the transportation sector (42, 43). For instance, Zhou et al. (44) studied the impact of different entry restriction policies on international air transport connectivity during COVID-19 and Zhou et al. (45) proposed a layered weighted network efficiency (LWNE) metric to study the vulnerability of the worldwide air transportation network in response to different levels of disruptions. Anke et al. (46) investigated the impact of SARS-CoV-2 on mobility behavior and found a profound impact on mobility behavior with decreases in public transport and increases in car usage, walking and cycling. Furthermore, they also found that lockdown in the behavioral changes was minimal, which suggested isolated differences between policies with and without lockdown. Readers can also refer to further studies about infectious spreading on various transportation systems at different scales, including studies by Muley et al. (47), Kutela et al. (48), Choi (49), Hu et al. (50), Zhao et al. (51), and Severo et al. (52).

### 2.3. Research gap

From the above review, we found that recovery simulation of road networks, including many other types of infrastructure networks, based on different scenarios have been discussed extensively. All of them have substantially contributed to how we might effectively assess the resilience of urban road networks, optimize their recovery, and reveal the spreading mechanism of infectious diseases on urban transportation infrastructure. However, studies on recovery simulations of road closures in the context of public health crises, such as COVID-19 on road networks, with a good comparative analysis between static recovery strategies and dynamic recovery strategies are still needed. As stated in the previous section, we address this gap with two clear objectives in this paper.

## 3. Methodology

### 3.1. The analytical framework and basic assumptions

**Figure 1:**
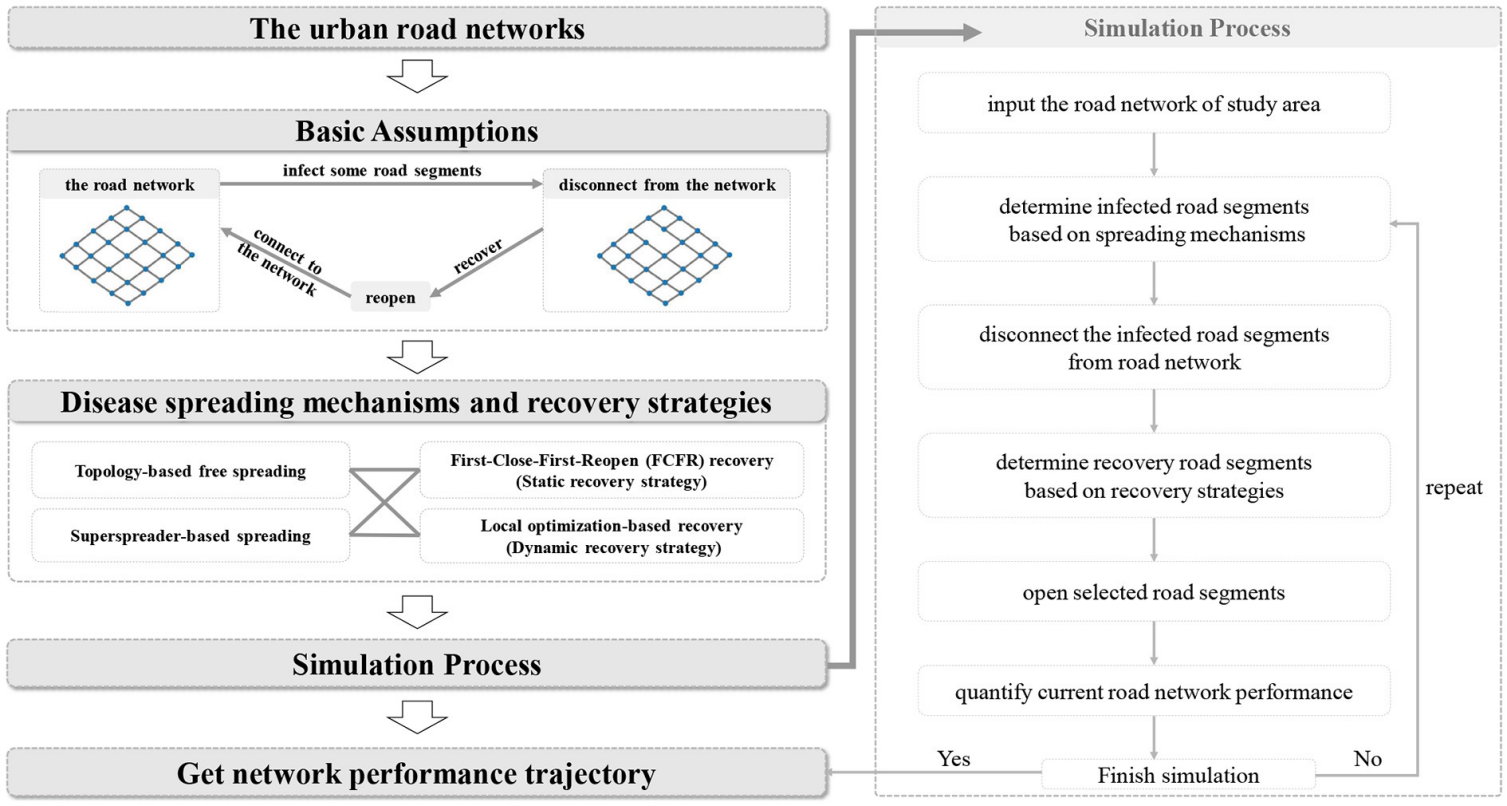
The proposed simulation-based analytical framework.

Figure 1 depicts the proposed simulation-based analytical framework that realizes the hybrid process of virus spread and policy interventions with a given road network (because most of the roads in the study area are dual-way roads, we therefore construct the network model as undirected). We design two spreading mechanisms (i.e., sporadic spreading and trajectory-based spreading) and two recovery strategies (i.e., static and dynamic recovery strategies) to simulate the spreading of infectious diseases and the recovery process of the network. Finally, we obtain the network performance profiles based on simulation outcomes and quantify the effectiveness of these two recovery strategies on road network resilience under different spreading mechanisms. Several basic, yet essential, assumptions should be presented as the prerequisites and cornerstones for the analysis.

1. ***In the simulation, we assume that all spreadings follow the topology of the road networks with a 100% infection rate***. This is based on the fact that a contagious disease spreads among populations due to intensive human mobility activities, and human mobility often hinges on the urban road topology. The discussion of (1) the actual spreading pathways from one individual to another and (2) the effect of dynamic population changes on epidemic spreading are not in our scope.
2. ***Unlike many previous studies, the affected objects in the simulations are not individual human beings or acting agents but road segments***. The infected road segments are assumed to be disconnected from the road networks. That is, once a confirmed positive case is identified along a particular road segment or in the community that is adjacent to a road segment, this segment is assumed infected, and as a response, this road segment is closed by the local government immediately; in other words, it is removed immediately from the network's accessibility due to its disconnection. Although this is a very strong assumption based on the “All-or-Nothing” principle (i.e., it is either full access or no access at all, limited or time-variant access is not considered), in real world practice, it is considered acceptable and realistic. For instance, for several countries that implement strict control policies, such as China, once a confirmed case is identified, the whole vicinity and community will be isolated and closed for full-screen sanitization, and thus, the roads around that area will be closed. Even in a less strict situation, if the vicinity is not completely isolated, people would still deliberately avoid those roads as soon as they know that there is a high risk of being infected around that particular vicinity, which is also roughly equivalent to road closure from a holistic perspective.
3. ***Disconnected road segments are assumed to be able to “reopen” after being treated with thorough sanitization or being identified as low-risk areas following restricted inspections (as an analog to the term “recover” in the typical failure-recovery paradigm)***. Moreover, in contrast to many previous studies assuming that recovery only starts after the completion of attacks/failures/removals, we let both the processes of closures and reopening happen simultaneously at each time step to simulate more realistic actions delivered by a city's emergency response team. In addition, we also assume that each road segment only experiences one closure in one round of simulation, i.e., we do not consider reinfections within those already-reopened segments.
4. ***Two types of spreading mechanisms are considered in this study, namely, the sporadic spreading mode and the trajectory-based spreading mode***. We assume that the former follows edge betweenness centrality and the latter has a certain associated infection range. Based on the previous study from Li et al. (38) and Tantrakarnapa et al. (53), the former spreading mode considers that the disease might sporadically occur at those highly populated spots where the density of roads and edge betweenness centrality are high, while the latter one mimics the negative impacts from superspreaders where the roads adjacent to (or within a certain range of) his or her trajectory will be considered high-risk areas that are very likely to be infected as well. Solid examples supporting this assumption in real world events can be found. For example, in January 2021, a super-spreader in Jilin Province, China, was identified to have had close contact with ~140 other individuals (54) and most of them later became confirmed positive cases, which directly incurred a city-level emergency response, including massive road closures and large-scale screening and testing. Super-spreaders may have no symptoms or slight symptoms, but they are highly infectious and unpredictable, i.e., scholars have dedicated considerable effort to decoding its fundamental mechanism, yet it still remains unclear (55).
5. ***The risk of infectious disease spreading based on a topological road network considers the following three scenarios:*** (1) all roads are assumed to be pedestrian accessible; (2) the commuting mode of super-spreaders on the road network could be both public transport trips or private car trips. The former may generate risks due to direct contact with other passengers. The latter may exacerbate risks due to random stops, such as staying at service stations and waiting at toll booths.

### 3.2. Road network performance

As mentioned in the literature review, various methods have been applied for road network performance quantification (56–59). Related topics are also popular in the field. In this study, one of the network connectivity indicators, network efficiency, is used to compute the network performance at each time step, as it is a well-defined indicator that considers both the network topology and the connection situation, which can directly link to the performance of the mobility flows on road networks. The network efficiency of the current road network *G* is defined in Equation (1). The equation computes the average reciprocal nearest distance among all the junction pairs in the network. As seen, the shorter the distance between nodes *i* and *j* is, the larger the network efficiency, and therefore, the better the connectivity of the road network.


(1) $$\begin{array}{l} E\left(G,t\right)=\frac{1}{n\left(n-1\right)}\underset{i\neq j\in G\left(t\right)}{\sum }\frac{1}{d\left(i,j\right)} \end{array}$$

where *E*(*G, t*) is the network efficiency of network *G* at time *t*. *d*(*i, j*) is the shortest distance between nodes *i* and *j* in the network structure at time *t*. *n* is the total number of nodes in network *G*.

To facilitate multilateral comparison and the calculation of the resilience in the later stages, we normalize the network efficiency to ensure that the initial network performance before any edge removals starts from 1 (i.e., 100% efficiency). The normalized network efficiency is determined by Equation (2), where *E*(*G*, 0) is the initial network efficiency of network *G*.


(2) $$\begin{array}{l} \bar{E}\left(G,t\right)=\frac{E\left(G,t\right)}{E\left(G,0\right)} \end{array}$$

### 3.3. Disease spreading mechanisms for simulations

#### 3.3.1. Sporadic spreading mode

The sporadic spreading mode considers that the infectious may sporadically occur at populated locations with high edge betweenness. In network theory, the edge betweenness centrality strongly indicates the centrality of each edge based on the shortest paths in all possible origin-destination (OD) pairs and describes the probability that an edge may be frequently passed by the OD movements, which on road networks indicates frequent traveler visits. Therefore, the betweenness-based propagation intuitively shows that crowded places have a higher infection probability (53). The edge betweenness centrality can be defined as Equation (3). Among all the shortest paths between all OD pairs on the road network, the more paths that pass the edge, the higher betweenness centrality the edge possesses.


(3) $$\begin{array}{l} B\left(e\right)=\underset{s\neq t}{\sum }\frac{\sigma_{s,t}\left(e\right)}{\sigma_{s,t}} \end{array}$$

where *s, t* ∈ *V* and *V* is the set of vertices in the network. σ_(_*s, t*) is the total number of shortest paths among *s* and *t*. σ_*s,t*_(*e*) is the total number of shortest paths from *s*, *t* pairs that pass edge *e*.

**Figure 2:**
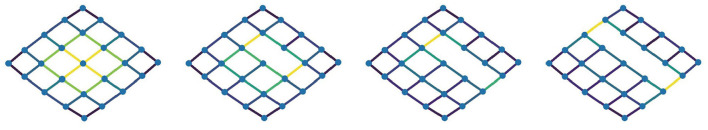
Network topology-based free spread model illustration (yellow: infected edge; green: travel path).

At each time step in this spreading mode, the road edges with top *s*(*t*) betweenness centrality values are set as infected. The betweenness centrality was recomputed at each time step to fully consider the dynamic influence of virus spread and updates of epidemic control policies. The spreading process is illustrated in Figure 2 by a grid network when the spreading speed is 1 edge/time step. At each time step, the edge with the highest betweenness centrality is identified as infected and therefore closed, which means this edge is removed from the network. Next, the betweenness centrality value of each edge is recomputed. The betweenness centrality value of each edge dynamically changes as the time step proceeds.

For the betweenness-based spreading mode, it is also essential to determine the spreading speed at each time step. Here, we select the most classic and widely applied SIR model. A typical SIR model can be depicted as Equations (4)–(6). For the basic theoretical implementation of this model, please refer to Hethcote (60) and Newman (61). This model describes the important temporal relationship among three groups during the epidemic propagation process, i.e., the susceptible group, the infected group, and the removed group (62). In this study, *S* indicates the group of edges that are susceptible to the virus in time step *t*. The number *S*(0), however, is smaller than the total number of edges in the graph to mimic the typical characteristics in the sporadic spreading mode. The variable *I* indicates the group of infected edges in time step *t*. The variable *R* indicates the group of recovered edges at time step *t*. *R*(0) equals 0 since no edge is reopened at the beginning of the propagation process.


(4) $$\begin{array}{l} \frac{\text{d}S\left(t\right)}{\text{d}t}=-aS\left(t\right)I\left(t\right) \end{array}$$

(5) $$\begin{array}{l} \frac{\text{d}I\left(t\right)}{\text{d}t}=aS\left(t\right)I\left(t\right)-bI\left(t\right) \end{array}$$

(6) $$\begin{array}{l} \frac{\text{d}R\left(t\right)}{\text{d}t}=bI\left(t\right) \end{array}$$

where *S* is the number of susceptible edges, *I* is the number of infected edges, and *R* is the number of reopened edges. *a* and *b* are two real, positive constant parameters used to control the model.

Based on the SIR model, the spreading speed at each time step can be inferred by Equation (7). The spreading speed equals the total number of infected and reopened road segments at time step *t* minus that of the last time step *t*−1.


(7) $$\begin{array}{l} s\left(t\right)=I\left(t\right)+R\left(t\right)-I\left(t-1\right)-R\left(t-1\right) \end{array}$$

where *s*(*t*) is the propagation speed at time step *t*. Correspondingly, the recovery speed *r*(*t*) at each time step can be determined based on the time differences of the total number of removed edges.


(8) $$\begin{array}{l} r\left(t\right)=R\left(t\right)-R\left(t-1\right) \end{array}$$

#### 3.3.2. Trajectory-based spreading mode

This mode is defined as the superspreader spreading mode in this paper. Super spread events have drawn increasingly high public attention in recent years (63). In this spreading mode, a superspreader's traveling path is first defined by assuming he or she would always go for the shortest distance to the destination (for simulating a shortest-path-based trip chain in daily commuting behaviors) and travel along this path with a predefined constant speed. At each time step, the surrounding road edges within a predefined given radius of the vicinity are identified to be high-risk segments that local authorities would close to prevent further cascading spreading, which might be caused by this particular superspreader in a worst case emergency response plan. Therefore, the spreading speed *s*(*t*) is coupled with and determined by the superspreader's traveling speed and vicinal road density (i.e., the faster the traveling speed and the higher the vicinal road density are, the more road segments might be infected and then closed at each time step).

**Figure 3:**
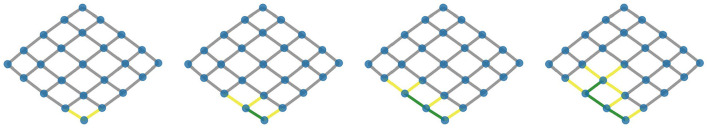
Trajectory-based cascading spread model illustration (yellow: infected edge; green: travel path).

Figure 3 illustrates the mechanism of the trajectory-based spreading mode when a superspreader travels from the bottom corner to the middle of the grid. For a better visualization, the infected (closed) road segments are colored rather than removed. The green lines indicate the superspreader's travel path, and the yellow lines denote the closed vicinal road segments. The breadth first search algorithm proposed by Eppstein (64) is used to determine the vicinal road segments at each time step in this set of simulations.

### 3.4. Recovery strategies

#### 3.4.1. First-close-first-reopen (FCFR) recovery (static recovery strategy)

**Figure 4:**
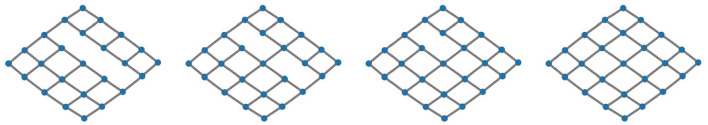
Trajectory-based cascading spread model illustration (yellow: infected edge; green: travel path).

The FCFR strategy, a common practice of recovery and maintenance schemes in previous studies and real world practices, is used as the benchmark for comparative analyses. As self-explained, this recovery strategy reopens the closed road segments based on their infection sequence; the first closed road segment will be treated and reopened first in the recovery process. Figure 4 shows an illustration of such a recovery process given the spreading mode from Figure 2. Although FCFR is the most widely applied and intuitive response principle in emergency events, it is obviously not a proactive approach as it does not dynamically consider the influence of the reopened road segment on the rest of the closed roads or the changes in the overall network performance. Thus, we refer to the FCFR recovery strategy as the static strategy here in this study.

#### 3.4.2. Local optimization-based recovery (dynamic recovery strategy)

**Figure 5:**
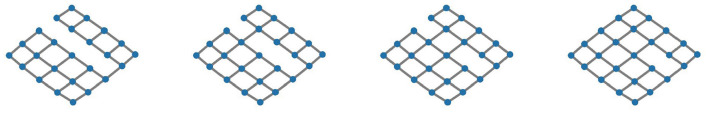
Illustration of the local optimization-based recovery process.

Given that the infections occurring on the road network follow either the spreading mode of Figure 2 or Figure 3, finding the global optimal reopening sequence of those closed segments is a nondeterministic polynomial (NP) problem. Especially as the number of infected edges increases, the complexity of this problem exponentially increased due to the increased number of combinations and system functionality quantification (65). Moreover, when the future propagation process remains unknown, making the global optimal recovery decision in the current time step is even more difficult. To ensure the simulations are both practically and computationally approachable, using the local optimization as the decision principle could be a simplified yet very feasible solution here. In this study, the dynamic recovery strategy is defined as reopening the road edges that can maximally improve current network performance (i.e., the network efficiency) compared to the last time step (Equation 9). Figure 5 illustrates the process of the local optimization-based dynamic recovery strategy when the reopening speed is 1 edge/time step.


(9) $$\begin{array}{l} argmax\left[p\left(t+1|a_{i}\right)-p\left(t\right)\right] \end{array}$$

where *p*(*t*) is the network performance at time step *t*, which is the efficiency in this paper (cross-referencing Equation 2), *p*(*t* + 1|*a*_*i*_) is the network performance at time step *t* + 1 after reopening the road segment *a*_*i*_, and *argmax* indicates that the optimization function is to find the best argument *a*_*i*_ that provides the maximum value from the function.

### 3.5. Simulation settings and pseudo codes

It has been proven that minor access roads play critical roles in maintaining the overall accessibility of road networks. Thus, we do not trim off those short and minor access roads in the selected network to simplify the topology for levitating computational burdens. In this vein, to facilitate the computation and ease the intense load, parallel computing and memory control processing techniques have been used in the iterative computations of the network efficiency in all simulations. The computing process is modified by the Python package, *Networkx* (64).

**Figure 6:**
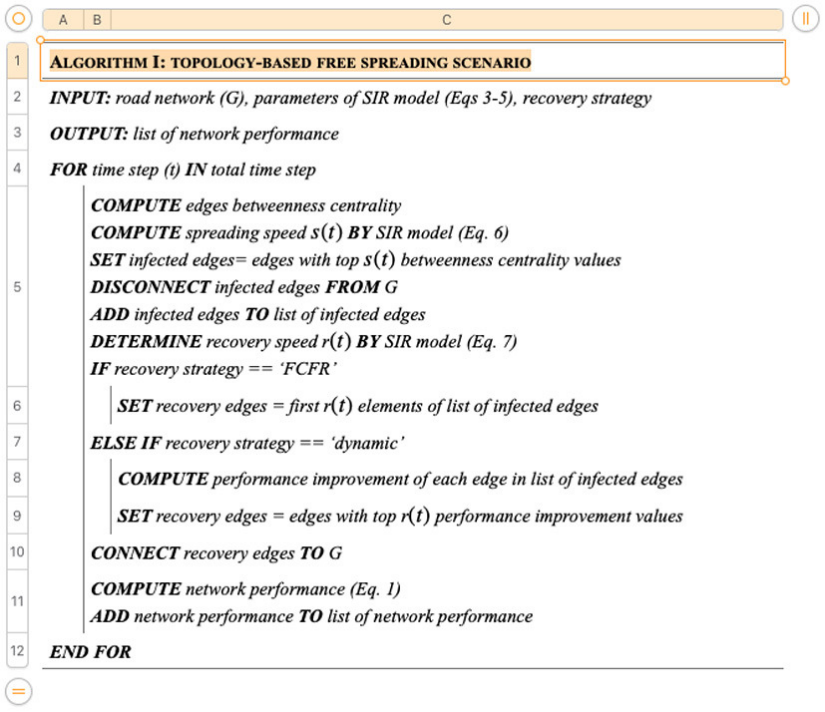
Algorithm I: Sporadic spreading scenario.

Two scenarios based on spreading mechanisms are simulated separately in this study to illustrate the influences of different emergency responses. Both recovery strategies are applied to these scenarios. Figure 6 represents the pseudocode for the sporadic spreading case, where the disease spreading speed and road reopening speed are determined by the SIR model. Hence, the input requires the road network information, the parameters from the SIR model, and the recovery strategy. The output is the resultant network performance which is quantified by Equation (1) at each time step.

**Figure 7:**
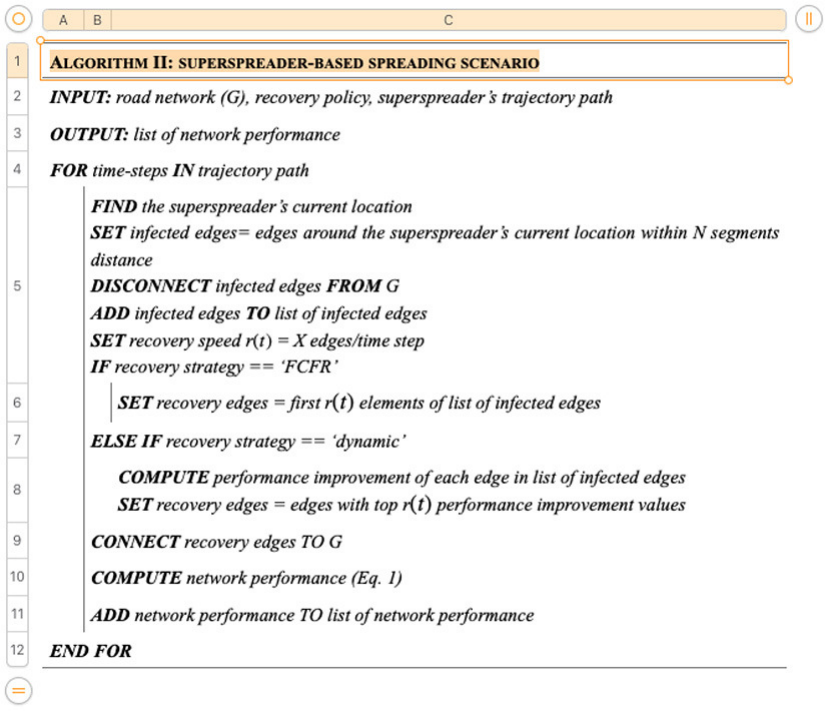
Algorithm II: Trajectory-based spreading scenario.

Figure 7 represents the pseudocode of the trajectory-based spreading simulations. The input requires the road network information, the recovery strategy, and a predefined trajectory path of the superspreader. The spreader is assumed to travel along the trajectory with a fixed traveling speed. At each time step, the location of the traveler along the trajectory is used to identify the vicinal road segments within a given range. Here, this range is set as within *N* segments, i.e., all segments that can reach the current superspreaders location within the distance of *N* linked road segments. Hence, the number of total infected road segments predominantly hinges on the local road density. In contrast to the first scenario, the reopening speed of the trajectory-based spreading simulations is set as a constant for the purpose of simplification and later for comparative analysis.

## 4. Case study

**Figure 8:**
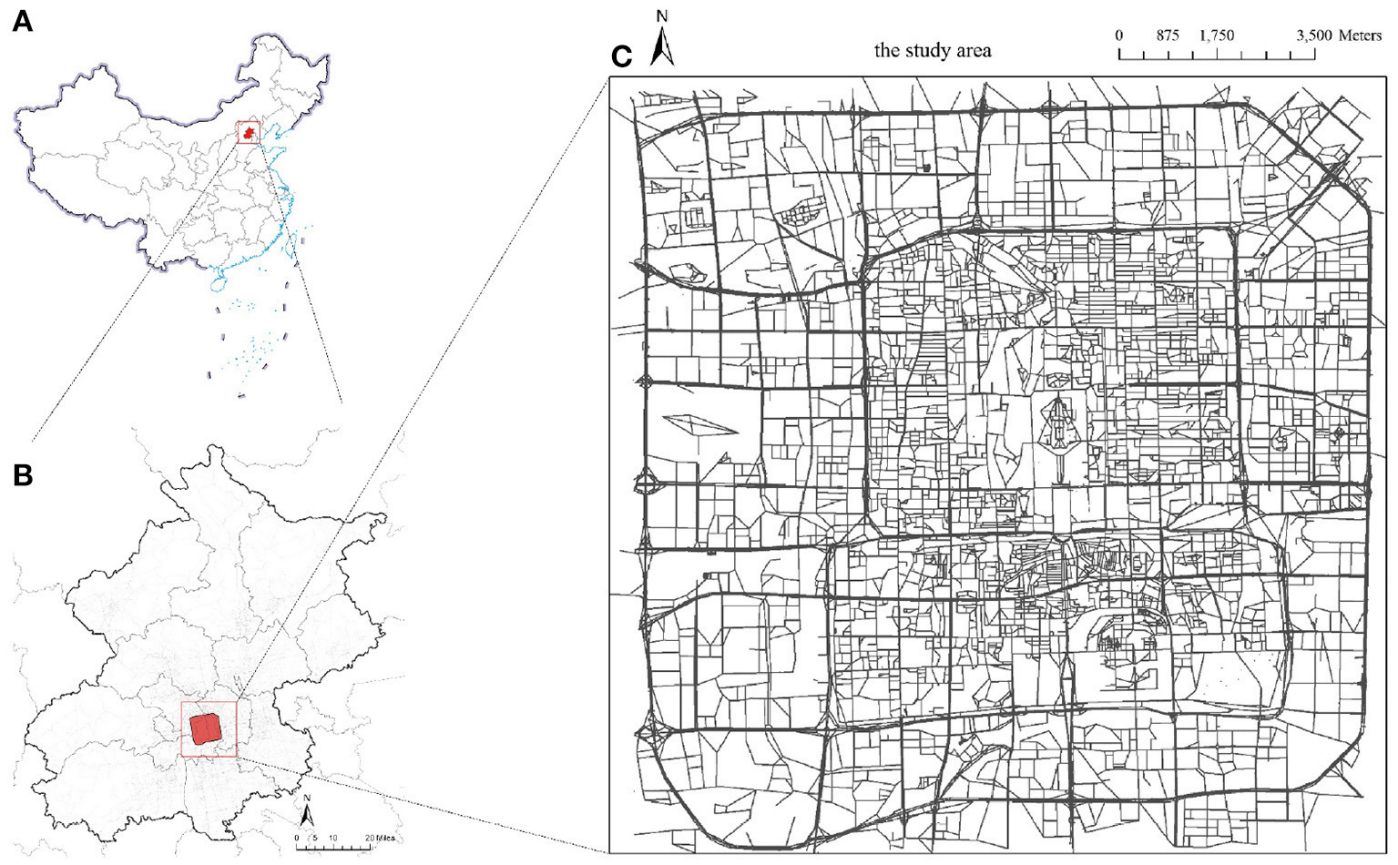
The study area. **(A)** China. **(B)** Beijing. **(C)** The study area.

Beijing, the capital city of China, was selected as an illustrative case study here. It is known as a representative megacity and ranked as one of the top 10 cities in the 2021 Global Cities Index (66). As a high-density megacity and an important hub of the Beijing-Tianjin-Hebei agglomeration, Beijing had a large population of 21.89 million permanent residents in 2020 and an unparalleled annual traffic volume (67). A virus outbreak in Beijing would lead to severe impacts on the management of urban operations and people's travel behaviors (68). However, Beijing suffered second wave of COVID spread in 2020, and there was no local transmission within 56 consecutive days under extremely strict control measures (69). Therefore, it is of practical importance to take Beijing as a case study to investigate how megacities should respond to the spread of the virus and learn from it. Here, the study area is confined within the inner third-ring road of Beijing (Figure 8). This study area covers the very core of its massive road network, including 16,008 junction nodes, 22,062 road segments, and a total road length of ~2,300 km.

**Figure 9:**
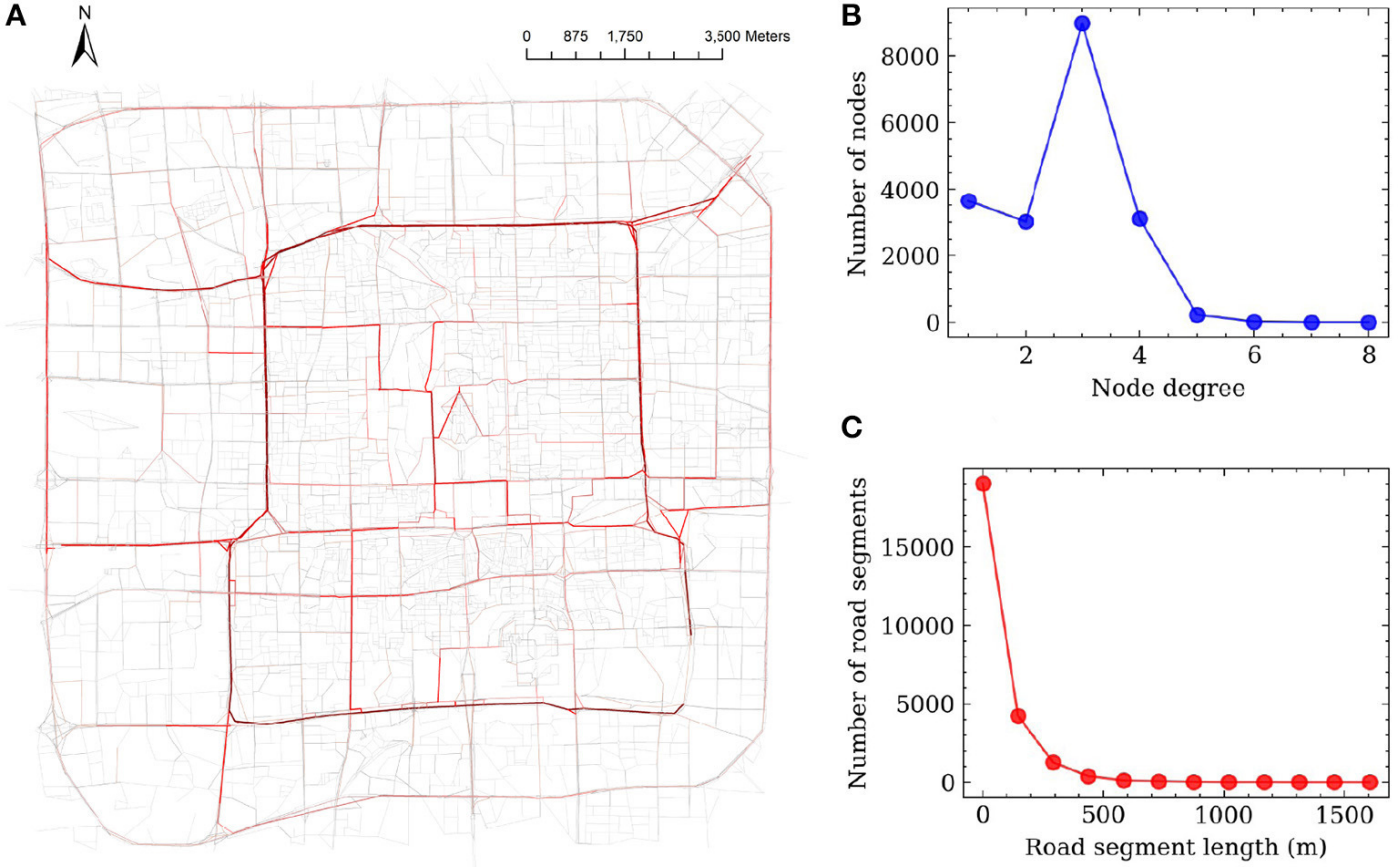
Node degree distribution of Beijing Road network. **(A)** Betweenness centrality degree. **(B)** Node degree distribution. **(C)** Road length distribution.

Figure 9 shows the descriptive analysis of the road network statistics. Figure 9A indicates the edges' betweenness centrality values when all the road segments are functional. The road segments with higher betweenness centrality are shown in red, where we can clearly see that those high-betweenness edges are concentrated around the center of the study area and are mainly trunk roads or major urban streets. Figure 9B displays the degree distribution of the nodes; most of the nodes have a degree of 3, which often represents a “T” shape junction in the topology (a node with degree 4 represents a normal four-way intersection), and this number is in line with the findings from previous studies. A few nodes' degree is larger than 4, which may be caused by the extra ramps on highways or roundabouts. From the road edge length distribution (Figure 9C), it is clear that most of the road segment lengths are <500 m, and a few road segments have lengths of over 1,000 m.

## 5. Results

### 5.1. Sporadic spreading scenario

**Figure 10:**
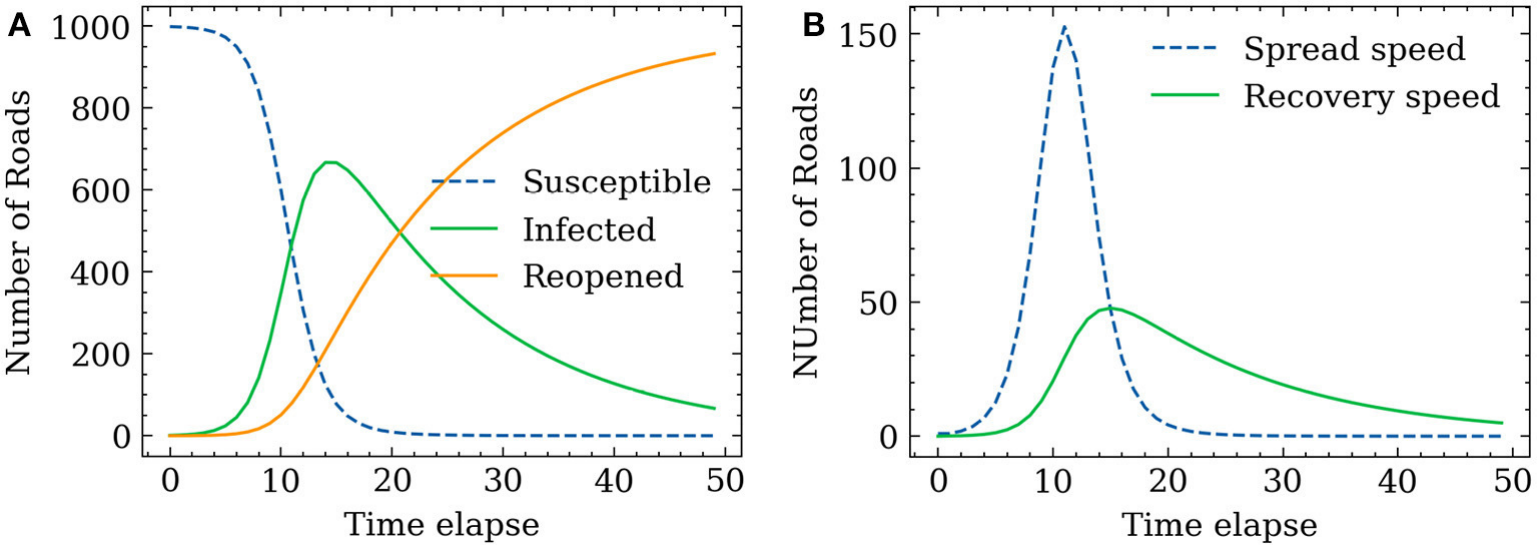
SIR model simulated propagation process. **(A)** Number of each group. **(B)** Propagation and recovery speed.

From the methodology, we utilize the SIR model to determine the spreading speed *s*(*t*) and recovering speed *r*(*t*) in this simulation scenario. Two parameters that control the SIR model, the infection rate *a* and recovery rate *b*, are selected from Cooper et al. (70) to imitate a real pandemic spreading process. The number of susceptible road segments is set as 1,000, which accounts for ~4.5% of the total road segments. *a* and *b* in Equations (3)–(5) are set as 0.35 and 0.035 based on the referred study (we reproduced the SIR simulation strictly following the instructions in the paper. Readers can refer to it for more details on the SIR model construction and simulation settings). The time-dependent evolving processes of the susceptible group, infected group, and reopened group are shown in Figure 10A. The results of the SIR model indicate that the number of susceptible road segments gradually decreases at the beginning as the disease begins to spread. After ~5 time steps, the gradient of the curve of infected road segments increases, and a large portion of the susceptible group has been infected. The simulation is ended at 50 time steps (to cover a long enough wave of epidemic strike). All the susceptible road segments are infected, but a small portion of road segments are not recovered at the end of the simulation. The corresponding spreading speed and recovery speed are shown in Figure 10B. The largest spreading speed occurs at ~12 time-steps, where most road segments are infected but the recovery process was just about to initiate, and then the spreading process almost finished after the 20 time-steps. As described in the study framework (cross-referencing Figure 1), the segments are dynamically closed and reopened based on the spreading speed and recovery speed. It should be noted that with the betweenness centrality of each edge being recomputed at each time step, the infected road segments at each time step can be different when using different recovery strategies.

**Figure 11:**
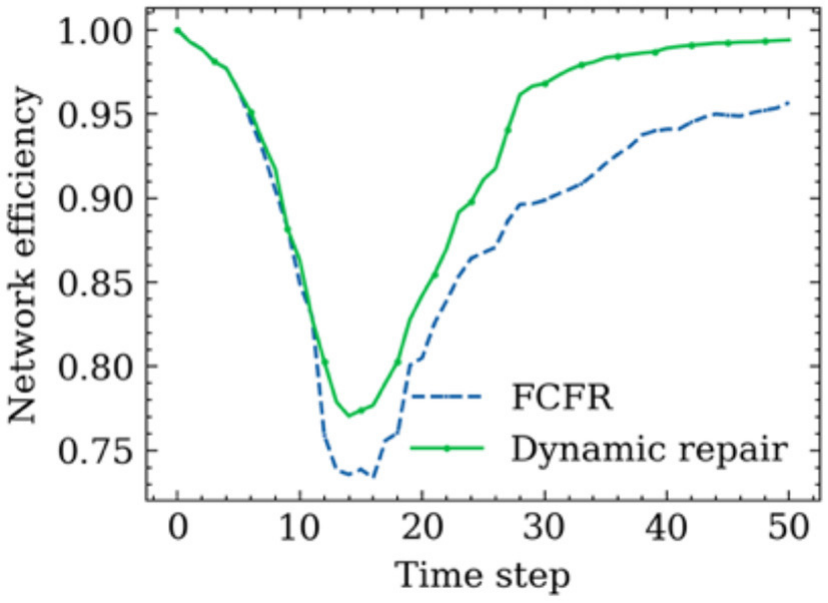
Hybrid influence of restriction and reopen influence of the different recovery models.

Figure 11 shows the road network performance curves in the context of the two proposed recovery strategies. A relatively similar network performance decrement trend between the FCFR recovery and dynamic recovery can be observed at the initial stage of spreading process. The discrepancy between the two became more obvious after ~10 time-steps, which corresponds to the increasing recovery speed as seen in Figure 10B. Although the network performance touches its lowest point at a similar time step regardless of the recovery strategy, the dynamic recovery clearly demonstrates a much higher performance value than the FCFR method, which indicates a better and more resilient performance by losing less total system functionality.

The advantage of using the dynamic strategy is even more critical during the later stage of the spreading process, and it can be observed that the dynamic recovery strategy almost achieves 100% of the original performance whereas the FCFR strategy only reaches ~95% of the pre-event performance level. Overall, the performance loss of the dynamic recovery strategy is only ~4.63, which is 9.1% of the total performance area, while that of the FCFR recovery strategy reaches 6.49 (12.7%), which again quantitatively confirms the advantages of the dynamic recovery compared to the FCFR recovery.

However, we can also see from the simulation result that the sporadic occurrence of disease can cause considerable harm to the road network as the performance level drops sharply within the first 10 time-steps. Because it is sporadic in space, it can occur so irregularly and widely separated in place that one single emergency response team might find it struggle to quickly react to those successive but very distant outbreak spots. Thus, to deploy an effective dynamic recovery strategy in this spreading mode, a centralized control center that can put multiple emergency response units into in-time actions should be considered. In practice, it is true that many cities establish centralized epidemic control centers in their local authorities.

### 5.2. Trajectory-based spreading scenario

#### 5.2.1. A simple non-commuting trip

**Figure 12:**
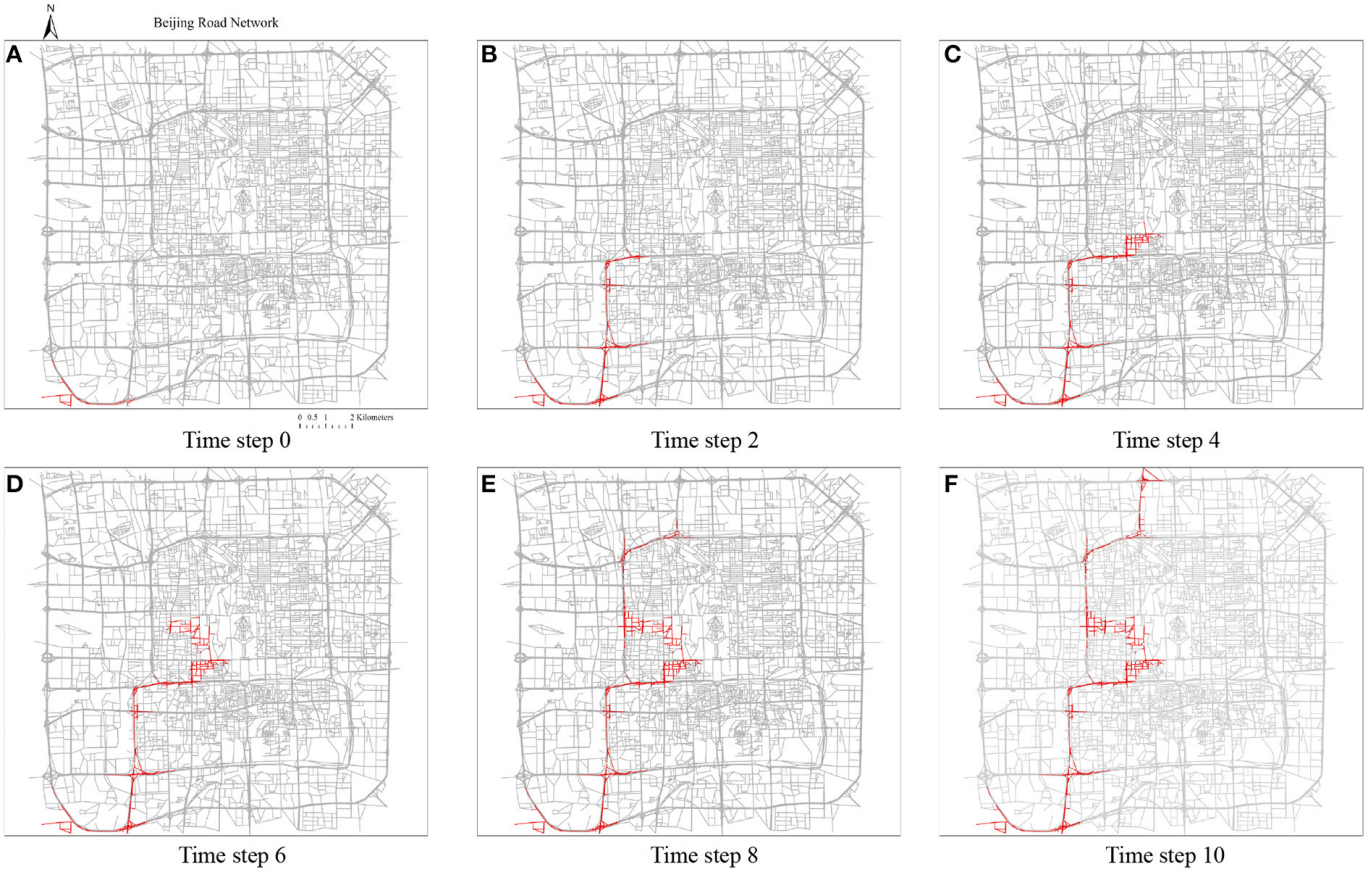
The trajectories of superspreader-based infections propagation. The traveling speed is set as 10 road segments/time step. The closest five segments adjacent to the traveler are assumed to be infected. The total moving time is 11 time steps. **(A)** Time step 0, **(B)** time step 2, **(C)** time step 0, **(D)** time step 6, **(E)** time step 8, **(F)** time step 10.

Because there could be no typical symptoms, a superspreader very often does not have self-awareness of being a superspreader until the ones infected by him/her are positively confirmed, causing this mode of spreading to be extremely difficult to control. Figure 12 demonstrates the superspreader's traveling path and infected road edges. For this randomly selected OD trip, the superspreader starts from the southern left corner, which is close to the location of the Beijing south high-speed railway station (a very crowded place with numerous commuters on weekdays), to the north of the study area (a place where commercial offices and residential neighborhoods are clustered). In this demonstrative case, the superspreader would pass the center of the road network for the shortest path from his or her origin to the destination, which is intuitively not optimistic for epidemic control as it is highly populated and the road density is also high in the city center. Consequently, this leads to more road segments being infected in the center area in this spreading scenario than on the previous scenario.

**Figure 13:**
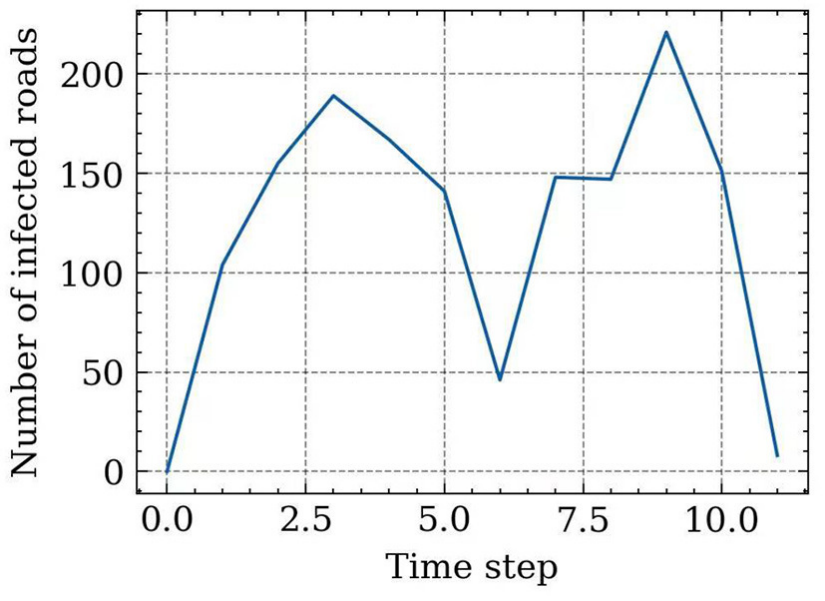
The number of infected roads at each time step of trajectory-based propagation.

Based on the demonstrative case in Figure 12, we set the recovery speed as 50 road segments/time step, and thus, the number of infected road segments at each time step (spreading speed) can also be inferred, which is shown in Figure 13. This curve demonstrates that the spreading speed is highly correlated with the road density in the network. The spreading speed increases when the superspreader travels from the southwest corner to the center of Beijing city. Around the 6th time step, the spreading speed decreases due to the shorter but denser road segments that are close to the center area. After that, the spreading speed increases again and eventually decreases after closing to the destination. A total of 1,477 road edges are infected during this spreading process.

**Figure 14:**
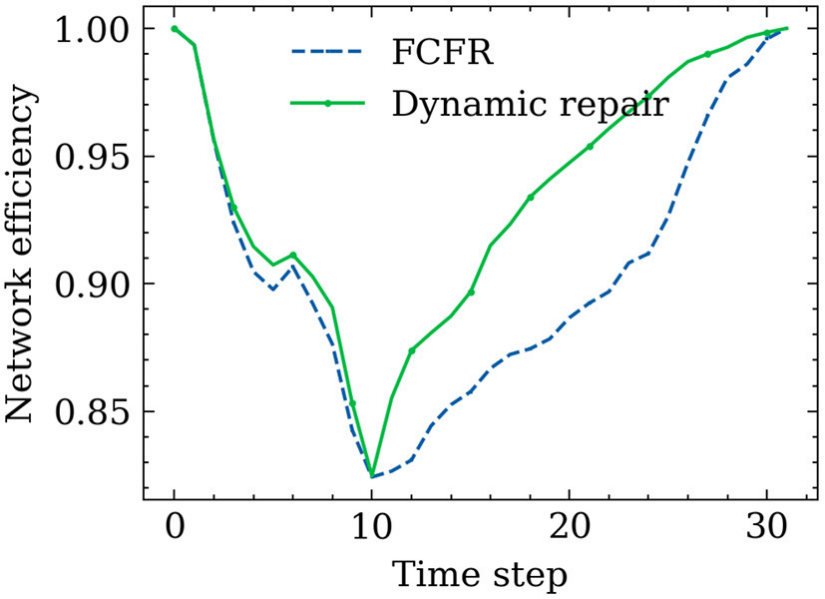
Trajectory-based recovery results.

Figure 14 shows the resilience curves of the network performance in this superspreader spreading scenario when implementing the proposed two recovery strategies. The total simulation time is longer than the travel time as the recovery process continues after the superspreader reaches the destination. Two similar-shaped performance drops can be observed in the first 10 time steps when using the FCFR recovery strategy and the dynamic recovery strategy, where a minor rebound occurred in the middle of the drop. One possible explanation for this minor rebound could be due to the large recovery speed compared to the superspreader's traveling speed (the effects from various recovery speeds are discussed in the following). Once the trip is completed, the merits of implementing the dynamic recovery strategy become more significant; it can be seen that the dynamic recovery strategy reopens those critical road segments much faster than the FCFR does, which again leads to a more resilient recovery process. Quantitatively, the performance loss in FCFR is ~3.98 (12.4%), whereas that figure in the dynamic recovery strategy is only 3.06 (9.6%), showing a 2.9% improvement for a single superspreader (Considering the large scale of the network in megacities, this improvement could already be pretty impressive). Furthermore, it only takes a second to realize that this single superspreader can cause almost the same level of damage as a series of sporadic spreads (as shown in Section 5.1).

#### 5.2.2. Sensitivity to the changes of trip trajectory

**Figure 15:**
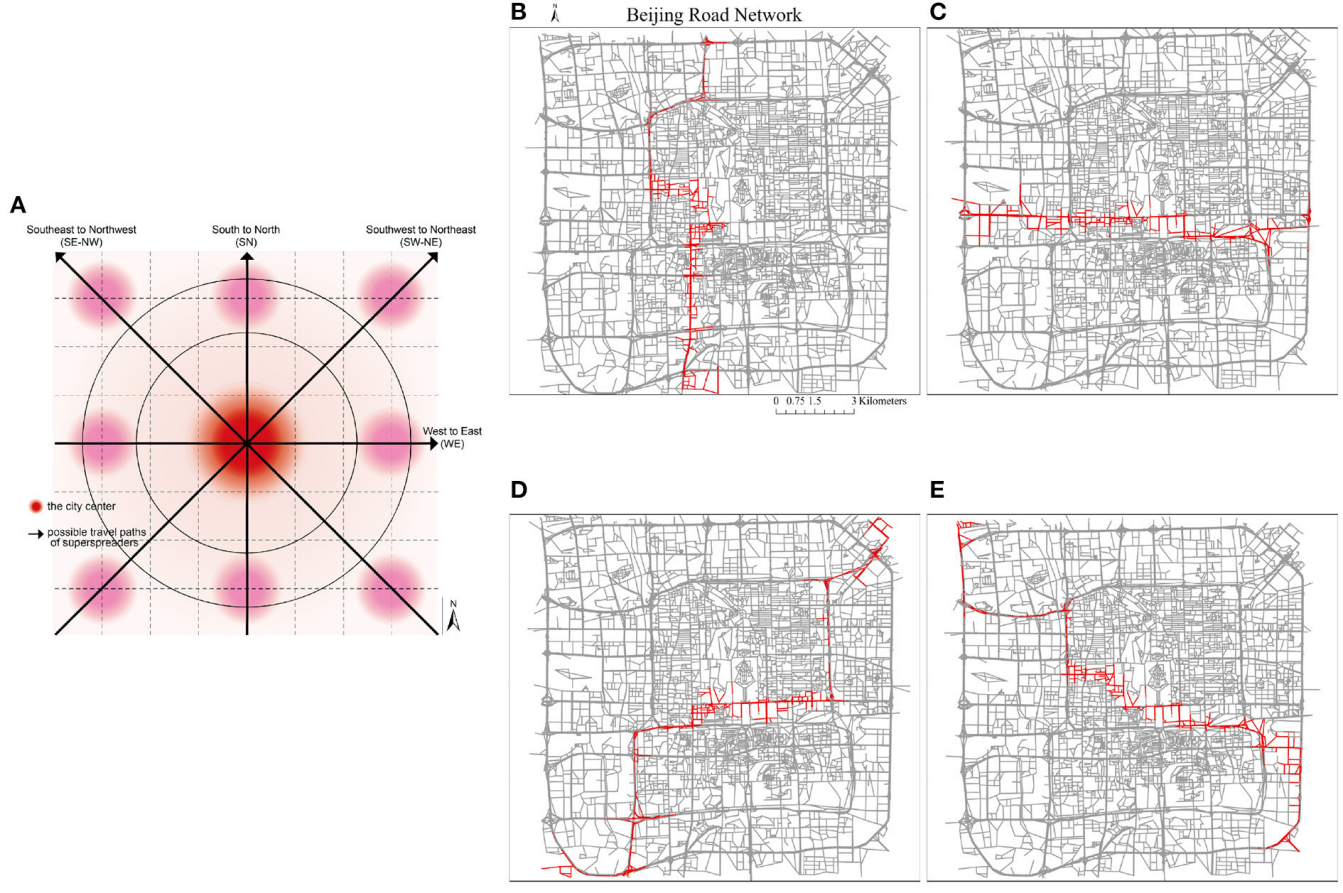
The trajectories with different orientations of a superspreader. **(A)** An illustration of possible traveling paths of superspreaders. **(B)** South to North. **(C)** West to East. **(D)** South-west to North-east. **(E)** South-east to North-west.

To test the robustness of the merits of the dynamic recovery strategy, another four scenarios with heterogeneous trajectory paths and recovery speed are designed and simulated. An illustration of the four trajectory paths with different orientations in the city is shown in Figure 15A. The traveling speed is kept at 10 road segments/time step. The infection range is considered as 3 segment distances in this round of simulation. As shown in Figures 15B–E, the travel directions from south to north (SN), west to the east (WE), southwest to northeast (SW-NE), and southeast to northwest (SE-NW) are simulated. The recovery speeds are varied from 50 road segments/time step to 5 road segments/time step to facilitate the comparative analysis, where Figure 15B with recovery speed of 50 road segments/time step, Figures 15C,D has a recovery speed of 10 road segments/time step and Figure 15E with recovery speed of 5 road segments/time step. The appropriateness of this parameter setting can be explained as follows: (1) because there were no similar studies as references, in this study, we perform several sets of trials based on the simulation settings, and it was found that the parameters selected above are optimal in terms of simulation accuracy and computation time; (2) considering the virus propagation speed and realistic medical resource allocation, an excessively fast recovery speed may reduce the benefits of optimal decision-making, so this study set the recovery speed from 50 road segments/time step to 5 road segments/time step; and (3) this study may provide a reference for the setting of these types of parameters in future studies.

**Figure 16:**
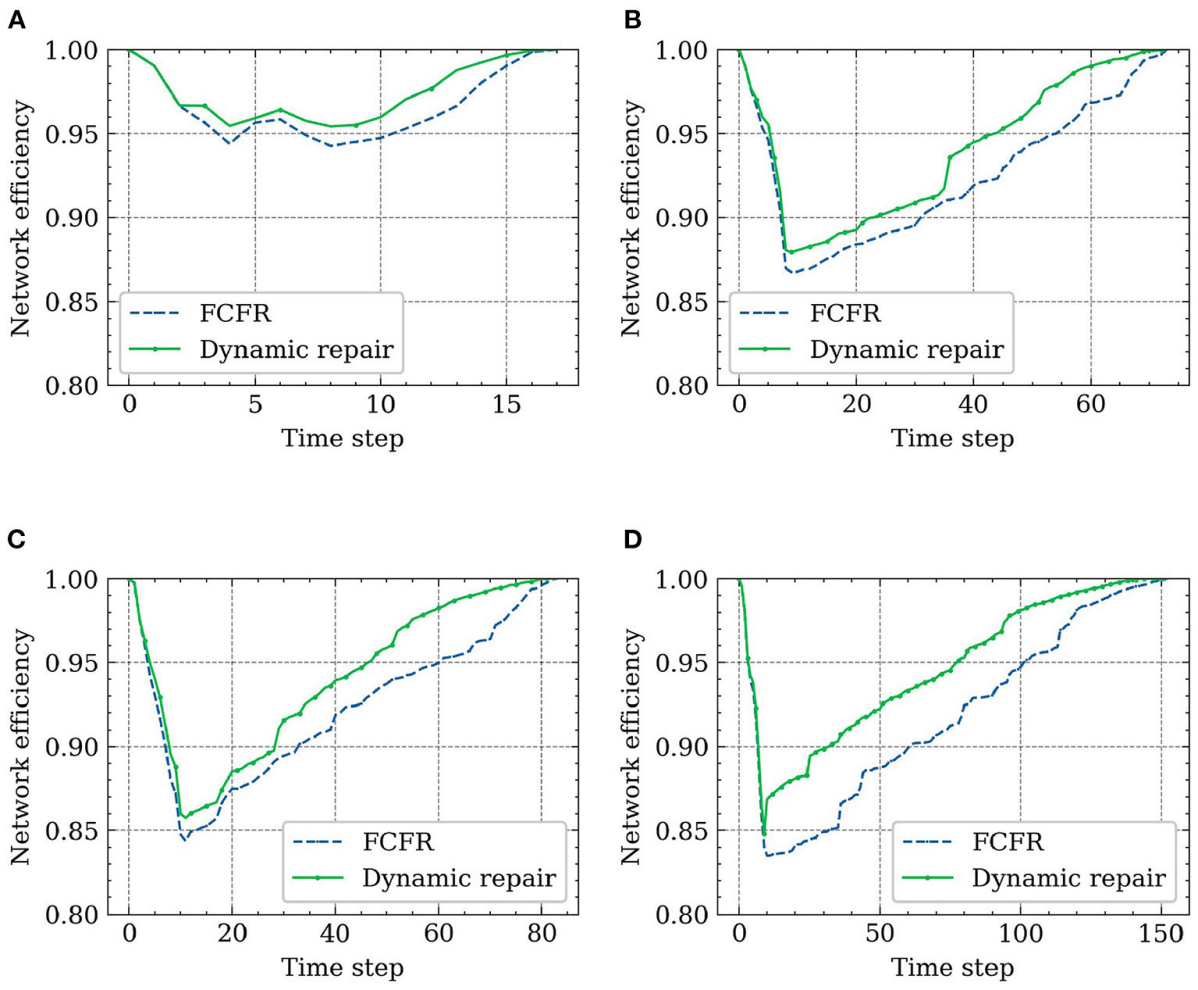
Final recovery results with different traveling paths and recovery speeds. **(A)** Recovery results of SN path (R = 50 roads/time step). **(B)** Recovery results of WE path (R = 10 roads/time step). **(C)** Recovery results of SW-NE path (R = 10 roads/time step). **(D)** Recovery results of SE-NW path (R = 5 roads/time step).

**Figure 17:**
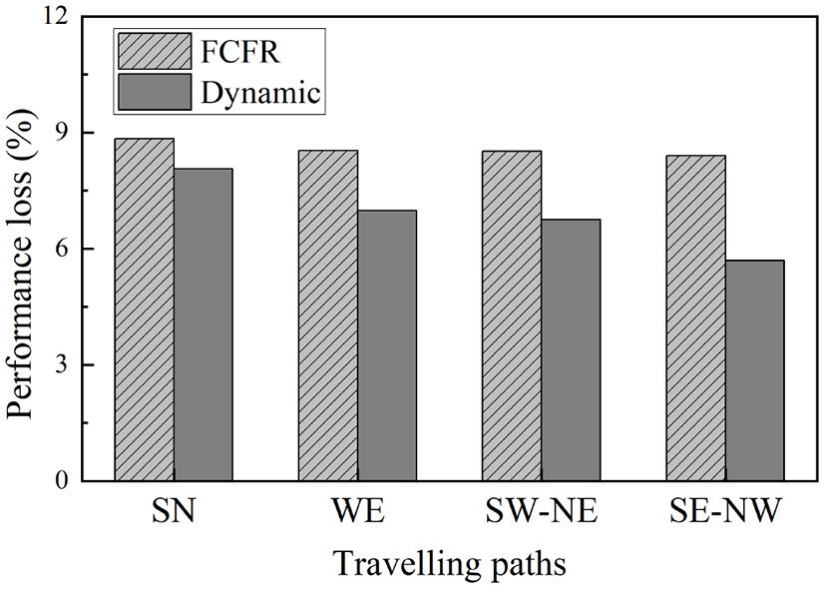
Performance loss with different traveling paths and recovery speeds.

Comparing the performance curves from each orientation (Figure 16), the minimum impact to the road network scenario is when the superspreader travels from south to north with a recovery speed of 50 road segments/time step, which is an intuitive observation as the faster the response and reopening speed, the less the negative influence caused by the superspreader (regardless of which recovery strategy), i.e., performance loss of 1.59 (8.8%) in FCFR and 1.45 (8.1%) in dynamic. It can also be seen that the influence of superspreaders on network efficiency from southeast to northwest is more severe than that from south to north, i.e., performance loss of 12.85 (8.4%) in FCFR and 8.71 (5.7%) in dynamic. It is likely that this case affects more segments and many of the infected segments are in the central region. Graphically, we can see that the superiority of adopting a dynamic recovery strategy can be observed in all simulated scenarios. Comparing the scenarios with respect to different recovery speeds (Figures 16A,B,D, the corresponding performance loss indices are shown in Figure 17), which indicates that the superiority of the dynamic strategy is more obvious when the recovery speeds are slow.

This comparison also indicates that a slower recovery speed leads to a greater performance loss, and thus, less resilient network functionality (Figure 17), which further corroborates that the negative impact on the road network caused by superspreaders could be more severe than expected. Thus, this mode of virus spread is the one that most attention should be given to in airborne epidemic emergencies, such as COVID-19. Numerically, even superspreaders behave differently according to various orientations and road densities in the simulations, the averaged performance loss from the FCFR recovery is ~6.98 (8.6%), while it is of only 5.25 (6.9%) from the dynamic recovery.

## 6. Discussion and implications

In terms of the comparison between the two recovery strategies, this study quantifies the considerable advantage of dynamic strategies over static FCFR strategies. Given that in many countries or regions it is not always possible for public health resources to be greatly reinforced in the short time of the spreading, thus, it is undeniably necessary to implement active, dynamic and strict epidemic prevention and control measures from early stage. This is intuitive, yet very often neglected in practice; observation again reiterates the importance of dynamically updating decision-making in fighting epidemics. In reality, this quick reaction often means a joined effort from different city authorities such as local public health authorities, land and transportation authorities, and emergency management authorities. Moreover, in the simulation, the dynamic recovery strategy has comparable advantages against trajectory-based spreading (the resilience of the dynamic recovery strategy is 3.6% higher than that of the FCFR recovery strategy). The same pattern observed in other testing cases indicates that this advantage of the dynamic recovery strategy is stable and generic. Meanwhile, we also found that the spreading speed of superspreaders is highly correlated with the road density (and orientations), which could imply that city managers should pay more attention to urban areas with denser roads from the perspective of epidemic prevention and design with extra care in urban street planning, especially in the post-pandemic era.

To reduce the risks of infection caused by super spreaders, it is effective to use smart and digital technologies for epidemic prevention and control, especially in tracking the trajectories of confirmed cases, analyzing crowded gathering hotspots and human mobility patterns, applying contactless transportation, etc. For example, using telecom technology and data analytics, Vodafone, a telecoms and technology service provider, created heatmaps to help government in Lombardy, Italy learn about population movements (71). In China, AI-powered autonomous vehicles are used to deliver medical supplies and necessities to hospitals or isolated communities under remote video monitoring, which helps minimize direct contact between people (72). The Hyderabad state in India used an automatic license plate recognition system based on advanced learning algorithms to monitor travel speeds to help the government enforce the driving restriction order (i.e., citizens should not drive more than 3 km from their homes) (73). In addition to engaging technologies, policy measures are critical for dealing with superspreaders in urban transportation, such as initiating traffic-calming schemes (low-traffic neighborhood) (74). In fact, as part of the COVID-19 response, traffic-calming trials have been fast-tracked in many cities across the UK. However, due to multiple factors in personal travel behaviors, it remains challenging to deal with superspreading cases. In China, some optimistic examples of successfully controlling superspreaders exist, yet with appalling costs and sacrifices associated with the painstakingly strict control measures, such as in-depth epidemiological investigations and high-resolution tracing of contact trajectory.

To ensure a resilient and efficient urban road network, although many factors contribute to the spread of the virus, promoting proactive action plans with dynamic strategic measures should be taken as a bottom-line attitude regardless of the spreading mode. With the increasing emergence of various new variants of the new coronavirus, COVID-19, threats will continue, and the discussion of epidemic control will continue to emerge as well. For urban mobility, this might also continue triggering a growing number of relevant debates in public in the future. For example, the traffic-calming scheme mentioned above that was originally proposed to support social distancing and actively respond to COVID spreading by transforming dense-street residential areas has recently been heavily criticized due to critical cited concerns over congestion, emergency access issues and increasing inequity in local communities. Based on the findings and discussion in this paper, several policies and practical implications for improving the resilience of urban roads can be summarized as follows:

- In response to the spread of highly infectious epidemics, such as COVID-19, on urban roads, it will be more effective to combine dynamic recovery strategies with continuous preventive strategies rather than adopting one-size-fits-all static solutions, and this often requires a joint effort from multiple local authorities.
- To prevent and minimize the spreading risk caused by super-spreaders, city managers should consider even more proactive measures and seek diverse assistance from cutting-edge digital tools to achieve multiobjective epidemic control, such as tracking trajectories, monitoring real-time traffic, strengthening vehicle mobility management (e.g., congestion hotspot monitoring) and hierarchical control schemes for risk-prone areas.
- To maintain a more resilient urban road network during the pandemic time, if without an in-time response and a proper dynamic recovery strategy to ensure quick restoration, it is essential to acknowledge the following before implementing any epidemic control measures: The measure (such as road closure) could have negative impacts on road network resilience and might lead to severe consequences on accessibility and efficiency. In some cases, it could even exacerbate and provoke new issues, such as social inequity. For decision- and policy-makers, this is particularly noteworthy and should be borne in mind throughout the whole decision-making and policy-framing process.

## 7. Conclusion

This paper performs a simulation-based comparative investigation of the four different combinations of epidemic spread and recovery processes and quantitatively studies their impact on the resilience of Beijing's road network. We defined two modes of spreading mechanisms (i.e., sporadic occurrence and trajectory-based occurrence) and comparatively analyze the performance of urban road networks under two recovery strategies, namely, the “First-Close-First-Reopen” (FCFR) recovery strategy and the dynamic recovery strategy, to quantitatively benchmark their heterogeneous effectiveness, which provides several critical implications for practitioners.

The results show that (1) in terms of negative impact, the superspreader can cause much worse consequences for the overall accessibility of urban road networks. Given the real-world pandemic control cases, sporadic spreading and trajectory-based spreading could occur in tandem if no proper action is taken; (2) the road density and centrality order, as well as the network's regional geographical characteristics, can significantly affect the spreading speed of the virus and introduce heterogeneity into the recovery processes; and (3) In terms of better recovery strategy, we confirmed the superiority of the dynamic strategy; it considerably outperforms the common practice (i.e.,the static FCFR strategy). In the sporadic spreading scenario, the performance loss of the dynamic recovery strategy is only ~4.63, which is of 9.1% of the total performance area, while that of the FCFR recovery strategy reaches 6.49 (12.7%). In the trajectory-based spreading scenario, the average performance loss from the FCFR recovery is ~6.98 (8.6%), while it is of only 5.25 (6.9%) from the dynamic recovery.

This study provides insightful policy and managerial implications for city managers and policy-makers, which could inspire new strategies in managing public health emergencies during and even after the COVID-19 crisis. Like many other simulation-based analyses, we also acknowledge several limitations of this study as potential caveats for future roll-outs. First, our findings on the sporadic spreading mode rely on the basic assumptions and the parameter settings of the SIR model. Some assumptions of this paper might need to be further examined using more empirical data. For example, one of these assumptions is that the reopened road segments are assumed not to be infected and closed again. Urban roads, of course, could also be reclosed again due to the recurrence of the virus. This setting can be tested in future work. Another assumption, i.e., a 100% infection rate, was made to simplify the simulation process and to capture the worst spreading scenario. In the future, comparative studies based on various infection rates can be tested. Second, as the authors are writing this paper, new COVID variants have been periodically emerging. For instance, a SARS-CoV-2 variant named ‘Omicron’ has spread from South Africa and it was suggested that people who previously had COVID-19 may be more likely to become reinfected with this variant (75). Thus, future work could consider simulating spreading with various levels of infection rates. Third, the travel paths of road users may not always be perfectly in line with the road topology. However, in megacities, such as Beijing, China, it is very common for pedestrians to walk on the streets and for drivers to park vehicles on roadside parking lots. Nevertheless, the uncertainty of human behaviors is not considered in our simulations and can be included in future explorations. Fourth, the betweenness centrality and network efficiency considered in this study are unweighted. However, pandemics can also occur in non-central areas. In future studies, we will consider assessing the resilience of road networks using weighted metrics, such as the weighted network efficiency mentioned in the paper by Zhou et al. (76). Finally, focusing on only one megacity case study from China could also be a shortcoming of this study. More cities with heterogeneous characteristics will be explored as additional case studies in future research.

## Data availability statement

Publicly available datasets were analyzed in this study. This data can be found at: https://www.openstreetmap.org/.

## Author contributions

Conceptualization, methodology, data curation, validation, and software: JT, XF, and QL. Writing—original draft, investigation, and writing—review and editing: JT, HL, XF, XY, and QL. Funding acquisition and supervision: XF and JT. All authors contributed to the article and approved the submitted version.

## Funding

This research was supported by the Start-up Funding for New Faculty at Peking University Shenzhen Graduate School (1270110033) and Guangdong Basic and Applied Basic Research Foundation (2021A1515110537).

## Conflict of interest

The authors declare that the research was conducted in the absence of any commercial or financial relationships that could be construed as a potential conflict of interest.

## Publisher's note

All claims expressed in this article are solely those of the authors and do not necessarily represent those of their affiliated organizations, or those of the publisher, the editors and the reviewers. Any product that may be evaluated in this article, or claim that may be made by its manufacturer, is not guaranteed or endorsed by the publisher.
